# Identification and functional analysis of *Wall-Associated Kinase* genes in *Nicotiana tabacum*

**DOI:** 10.3389/fpls.2025.1543437

**Published:** 2025-02-05

**Authors:** Ling Li, Linggai Cao, Jintao Li, Zhiqiang Zhang, Jie Liu, Zhongying Ren, Jie Zhang, Rengang Wang, Yangfan Miao, Shizhou Yu, Wei Li

**Affiliations:** ^1^ Zhengzhou Research Base, State Key Laboratory of Cotton Bio-breeding and Integrated Utilization, School of Agricultural Sciences, Zhengzhou University, Zhengzhou, China; ^2^ Molecular Genetics Key Laboratory of China Tobacco, Guizhou Academy of Tobacco Science, Guiyang, China; ^3^ State Key Laboratory of Cotton Bio-breeding and Integrated Utilization, Institute of Cotton Research, Chinese Academy of Agricultural Sciences, Anyang, China

**Keywords:** wall-associated kinase, tobacco, evolutionary, expression analysis, stress response

## Abstract

**Introduction:**

Wall-associated kinases (WAKs) are pivotal in linking plant cell walls to intracellular signaling networks, thereby playing essential roles in plant growth, development, and stress responses.

**Methods:**

The bioinformatics analysis was employed to identify WAK genes in tobacco. The expression levels of *NtWAK* genes were assessed by qRT-PCR. The subcellular localization of WAK proteins was observed in tobacco cells and Arabidopsis protoplasts. Kinase activity of the WAK proteins was evaluated through *in vitro* assays.

**Results:**

We conducted a comprehensive genome-wide identification and analysis of the *WAK* gene family in tobacco (*Nicotiana tabacum*). A total of 44 *WAK* genes were identified in the tobacco genome, which were further classified into three distinct groups. Phylogenetic analysis comparing tobacco WAKs (NtWAKs) with Arabidopsis WAKs (AtWAKs) revealed species-specific expansion of these genes. The WAK proteins within each group displayed similar gene structures and conserved motif distributions. Promoter region analysis indicated that cis-elements of *NtWAK* genes are primarily involved in regulating plant growth and development, phytohormone signaling, and stress responses. Expression profiling under NaCl, PEG, and ABA treatments suggested that certain *NtWAK* genes may play key roles in modulating responses to abiotic stress. Three-dimensional structural predictions and subcellular localization analysis showed that NtWAK proteins from the three subgroups exhibit high cytoplasmic similarity and are primarily located to the plasma membrane. Kinase activity assay confirmed that they possess phosphorylation activity.

**Discussion:**

This study represents the first genome-wide analysis of the WAK gene family in *N. tabacum*, laying the groundwork for future functional investigations.

## 1 Introduction

Plants rely on intricate signal perception and regulatory mechanisms to respond to internal and external stimuli during growth, development, and adaptation to environmental stresses (Wolf, 2017). Receptor-like kinases (RLKs) are proteins located on the cell membrane that detect external signals and initiate intracellular signaling pathways. They play crucial roles in plant growth, development, and immune responses (Afzal et al., 2008; Dievart et al., 2020). Wall-associated kinases (WAKs), a family of receptor-like kinases localized to the plasma membrane, play a pivotal role in this process. Generally, a typical WAK protein comprises an extracellular galacturonan-binding (WAK_GUB) domain, a transmembrane domain, and a cytoplasmic protein kinase catalytic domain (Kanneganti and Gupta, 2008). Their extracellular domains tightly interact with the cell wall, while their intracellular kinase domains transmit signals, forming a crucial bridge between the cell wall and intracellular signaling networks (Anderson et al., 2001).

The *WAK* gene family was initially identified in Arabidopsis (He et al., 1999), and subsequent studies have demonstrated its significant roles in plant growth and development, regulating cell expansion, responding to environmental stresses, and defending against pathogens (Cosgrove, 2001; Wagner and Kohorn, 2001; Stephens et al., 2022). WAKs play a crucial role in plant growth and development by regulating cell expansion. Tomato SlWAKL2 and its homologous gene AtWAKL14 in Arabidopsis bind to pectin and oligogalacturonides, and positively regulate the development of vascular tissue (Ma et al., 2024). OsWAK11 monitors changes in pectin methy-lesterification within the cell wall and directly phosphorylates the brassinosteroid (BR) receptor OsBRI1 to regulate cell elongation in rice (Yue et al., 2022). In addition, WAKs play a role in stress response by sensing and transmitting external signals, regulating plant defense mechanisms and adaptability, helping plants cope with various environmental stresses. In tomato, the disruption of the *SlWAK1* gene impairs salt tolerance throughout the plant’s life cycle, primarily due to its failure to maintain osmotic homeostasis (Meco et al., 2020). In cotton, *GhWAKL26* may positively regulate salt tolerance by modulating the expression of ion transport-related genes, thereby altering the intracellular levels of Na^+^ and K^+^ (Gao et al., 2024). The Arabidopsis WAKL4-NRAMP1 regulatory module actively detects and responds to toxic environmental cadmium (Cd), effectively reducing its uptake (Yuan et al., 2024). GhWAK7A regulates cotton resistance to fungal diseases by interacting with the chitin recognition receptor complex GhCERK1-GhLYK5 in cotton (Wang et al., 2020).

*WAK* genes have been extensively studied in various plants, such as rice (Zhang et al., 2005; de Oliveira et al., 2014), cotton (Zhang et al., 2021, 2023), wheat (Guo et al., 2021; Du et al., 2024), and *N. benthamiana* (Zhong et al., 2023). Tobacco (*N. tabacum*), an important economic crop and model plant, possesses a complex genome resulting from polyploidization between two diploid progenitors (*N. sylvestris* and *N. tomentosiformis*) (Wang et al., 2023). This genomic complexity underpins the diversity and redundancy of functional gene families, particularly the multifunctional *WAK* gene family. However, their composition, structural characteristics, and potential functions in *N. tabacum* remain largely uncharacterized and require systematic investigation.

This study aims to systematically identify the *WAK* gene family in *N. tabacum* through a comprehensive genome-wide analysis, investigating their evolutionary relationships, chromosomal distributions, gene structures, conserved motifs, promoter *cis*-regulatory elements, and subcellular localization. Additionally, by analyzing gene expression patterns, the potential roles of *NtWAK* genes in stress responses will be explored. The findings will provide a foundation for understanding the biological significance of the *WAK* gene family in tobacco and their potential applications in crop improvement.

## 2 Materials and methods

### 2.1 Plant materials and stress treatments

The tobacco cultivar K326 *(Nicotiana tabacum* L., cv. Kentucky 326) was utilized to investigate gene expression under various stress conditions. Seeds were germinated in a soil mixture (vermiculite:humus = 1:1) and grown in a greenhouse at 22°C with a 16 h/8 h light/dark photoperiod for eight weeks. When the seedlings reached the six-leaf stage, uniform and healthy plants were selected for treatment. The experiment included four treatments: control (CK), 300 mM NaCl, 30% PEG-6000, and 50 μM abscisic acid (ABA) spray, with six sampling time points (0, 1, 3, 6, 12, and 24 h post-treatment). Among these, NaCl and PEG treatments were applied via root irrigation, while ABA was applied via foliar spraying. The experiment included three biological replicates for each treatment. Leaf samples were collected and stored at -80°C for RNA extraction. Primers are listed in **Supplementary Table S1**.

### 2.2 Identification of *WAK* genes in tobacco

The protein sequences of the Arabidopsis WAK family members were retrieved from the TAIR database (https://www.arabidopsis.org/) and used as query sequences (Verica and He, 2002). BlastP searches with an E-value threshold of 10^−5^ were conducted against the tobacco genome (K326, https://solgenomics.net/organism/Nicotiana_tabacum/genome) (Edwards et al., 2017) to identify candidate tobacco *WAK* genes, followed by redundancy removal from the output sequences. All candidate protein sequences were then submitted to the HMMER website for the identification of the conserved protein kinase (PK) domain (PF00069) and the wall-associated receptor kinase galacturonan-binding (WAK_GUB) domain (PF13947) (Potter et al., 2018; Zhang et al., 2021). The ProtParam tool (https://web.expasy.org/protparam/) was used to calculate the theoretical molecular weight (MW) and isoelectric point (pI) of each encoded protein (Zhang et al., 2019). CELLO v.2.5 (http://www.csbio.sjtu.edu.cn/bioinf/Cell-PLoc-2/) was used to predict the subcellular localizations (Yu et al., 2014).

### 2.3 Phylogenetic relationship, gene structure and conserved motif analysis

A multiple sequence alignment of WAK proteins from tobacco and Arabidopsis was performed using ClustalX 2.1 (Larkin et al., 2007). The aligned sequences were subsequently used to construct a neighbor-joining (NJ) tree with 1000 bootstrap replicates, using MEGA 7.0 software (Kumar et al., 2016). Genome annotation files provided the chromosomal locations and exon-intron structures of the identified *WAK* genes. The MEME tool (http://meme-suite.org/tools/meme) was employed to predict conserved motifs within each WAK protein, with the maximum number of motifs set to 12 and other parameters maintained at default settings (Bailey et al., 2009). The final result was visualized by TBtools (Chen et al., 2020).

### 2.4 Prediction of *cis*-acting elements

A 2000 bp region upstream of the coding sequence (CDS) of each tobacco *WAK* gene was retrieved from the tobacco genome to serve as the promoter sequence. *Cis*-acting elements were predicted using the PlantCARE database (http://bioinformatics.psb.ugent.be/webtools/plantcare/html/) (Lescot et al., 2002). The types, number, and positions of these elements were then quantified and visualized using TBtools software (Chen et al., 2020).

### 2.5 qRT-PCR analysis

Total RNA was extracted from the samples using the plant RNA kit (Omega Bio-Tek, USA). cDNA synthesis was performed with the StarScriptII RT Mix with gDNA Remover kit (GenStar, China). Gene expression levels under drought, salt, and ABA treatments were quantified by quantitative real-time PCR (qRT-PCR) using the primers listed in the **Supplementary Table S1**. qRT-PCR was conducted on a LightCycler480 system (Roche, Basel, Switzerland) with SYBR^®^ Premix Ex Taq™ II (TaKaRa, Dalian, China). The amplification protocol included an initial denaturation at 95°C for 30 s, followed by 40 cycles of 95°C for 3 s and 60°C for 30 s. Each reaction was performed in triplicate, and the data were analyzed using the 2^−ΔΔCT^ method (Livak and Schmittgen, 2001), with three biological replicates included in the analysis.

### 2.6 Three-dimensional structures and subcellular localization

The three-dimensional structures of NtWAK11, NtWAK32, and NtWAK41, predicted by AlphaFold, were retrieved from the UniProt database (https://www.uniprot.org/) through a homology search (Jumper et al., 2021; Varadi et al., 2022). The resulting structural models were visualized using PyMOL software (DeLano, 2002; Liu et al., 2024). Specific primers for *NtWAK11*, *NtWAK32*, and *NtWAK41* were designed using SnapGene v6.0.2 software for PCR amplification, and sequencing was verified by Sangon Biotech (Shanghai, China). The target gene without a stop codon was cloned into the *p*CAMBIA2300-GFP vector and introduced into *Agrobacterium tumefaciens* strain GV1301. The Agrobacterium suspension, containing either the recombinant or empty vector, was injected into the abaxial surface of 6-week-old tobacco leaves. After a 24-h dark incubation followed by 24 h of standard culture, protein localization was observed using Laser Scanning Confocal Microscopy (LSCM) (FV 1200, Olympus, Japan) with excitation at 488 nm. In addition, the recombinant plasmids were introduced into Arabidopsis protoplasts using the Arabidopsis Protoplast Preparation and Transformation Kit (Coolaber, Beijing, China). Protoplasts expressing the GFP fusion proteins were subsequently analyzed using LSCM.

### 2.7 Kinase activity *in vitro*

The *in vitro* kinase activity was quantified using a combination of luminescent detection and a luciferase assay to measure the remaining unreacted ATP, thereby enabling real-time monitoring of the kinase reaction. In this method, the luminescent signal is directly proportional to the ATP concentration in the solution and inversely correlated with the level of kinase activity (Wang et al., 2021). The kinase reaction buffer (10×) (500 mM Tris-HCl, pH 7.5, 100 mM MgCl_2_, and 10 mM DTT) was incubated with 40 μg/mL His-tagged NtWAK11, NtWAK32, and NtWAK41 kinases. The reaction was initiated by the addition of 0.45 μM ATP and 50 μM MBP substrate. Kinase activity of phosphorylated serine/threonine proteins was measured using the Kinase-Lumi™ Luminescent Kinase Assay Kit (Beyotime Biotechnology Company, Shanghai, China) according to the manufacturer’s protocol. After incubating the reaction at room temperature for 0, 5, 10, 15, 20, and 30 min, 50 μL of Kinase-Lumi™ chemiluminescent kinase detection reagent was added to each sample well and thoroughly mixed. Luminescence was measured using the chemiluminescence module of a full-wavelength multi-functional microplate reader (TECAN SPARK 10M, Switzerland).

## 3 Results

### 3.1 Identification of *WAK* genes in *N. tabacum*

**Figure 1:**
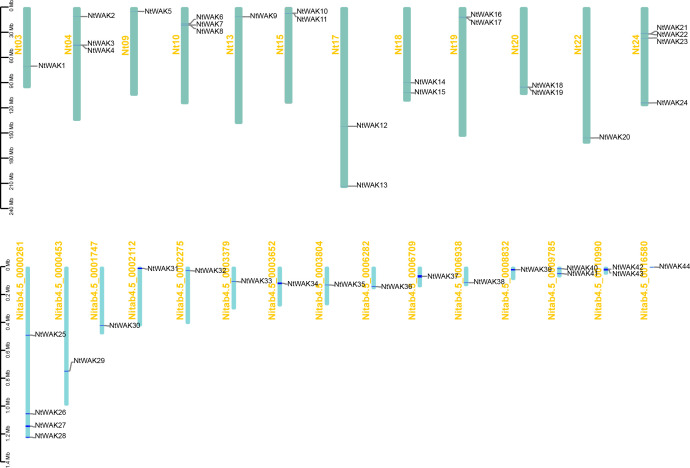
The localization of NtWAK family members on tobacco chromosomes. The bar graph shows chromosomes. Chromosome numbers are shown on the left and gene names on the right. The scale represents the length of the chromosome (Megabase, Mb).

Arabidopsis WAK protein sequences were used as query sequences in local BlastP searches to identify potential WAK family members in the tobacco genome. Candidate proteins were subsequently validated using HMMER to confirm the presence of the conserved PK domain (PF00069) and the WAK_GUB domain (PF13947) (**Supplementary Figure S1**). A total of 44 *WAK* gene family members were identified in tobacco using available genomic data (**Supplementary Table S2**). These genes were designated *NtWAK1*–*NtWAK44* based on their chromosomal positions. Among the 44 identified *NtWAK* genes, 24 were located on the chromosomes, while the remaining 20 were mapped to unassembled scaffolds (**Figure 1**). The distribution of *NtWAK* genes in tobacco was found to be uneven across 12 of the 24 chromosomes. Specifically, only one *WAK* gene was identified on chromosomes 3, 9, 13, and 22. In contrast, two *WAK* genes were located on chromosomes 15, 17, 18, 19, and 20, while chromosomes 4 and 10 each contained three. Chromosome 24 exhibited the highest gene density, with four *NtWAK* genes. Physicochemical analysis of the tobacco WAK proteins revealed that the protein length varied from 318 to 2434 amino acids, with theoretical isoelectric points ranging from 5.53 to 8.77. The molecular weights of the proteins ranged from a minimum of 35.82 kDa to a maximum of 274.48 kDa. The subcellular localization prediction results showed that most NtWAKs are localized to the plasma membrane (**Supplementary Table S2**).

### 3.2 Phylogenetic analysis of WAK genes

**Figure 2:**
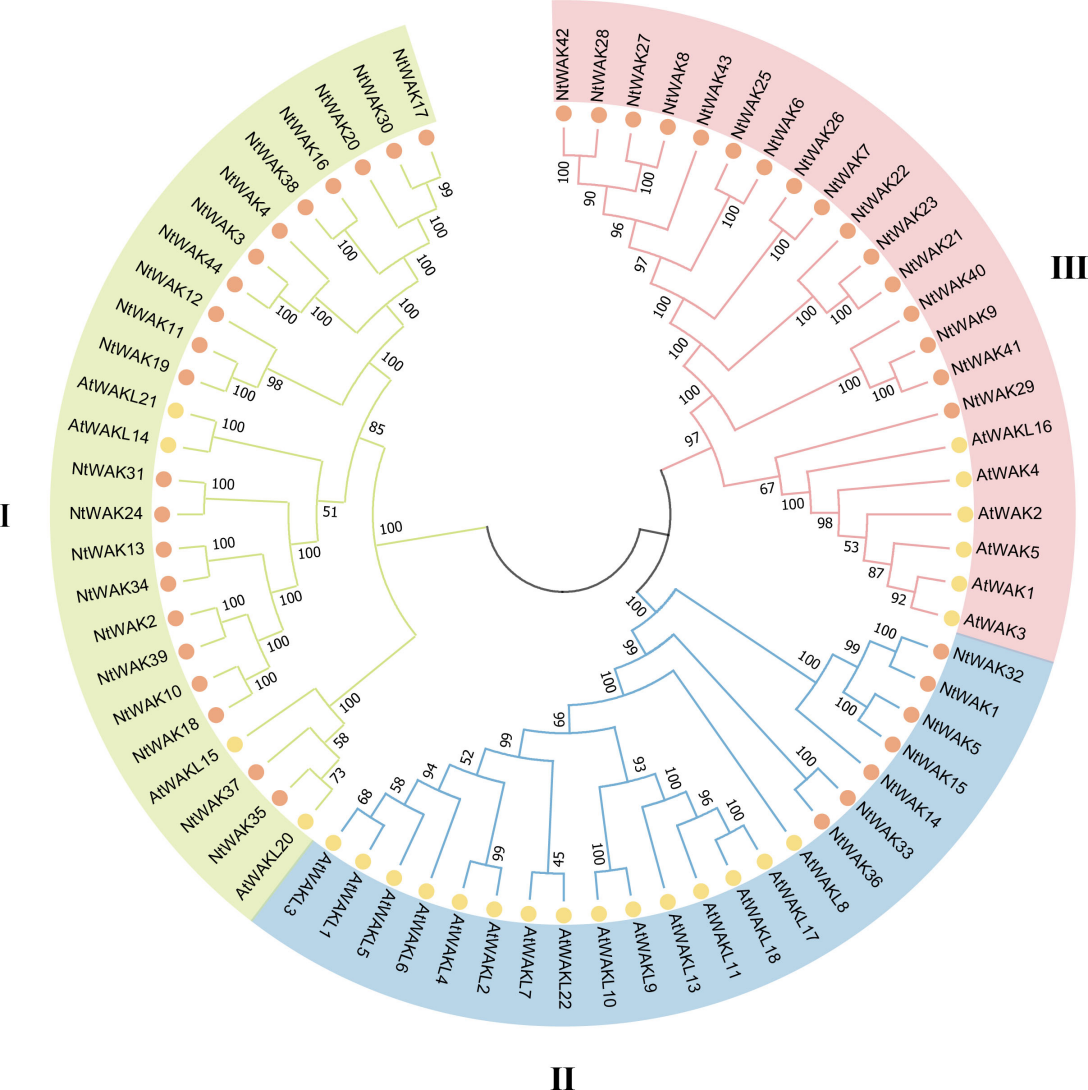
Phylogenetic analysis of WAKs. The phylogenetic tree was generated using the WAKs from *Arabidopsis* and *N. tabacum* with the neighbor-joining method in MEGA 7.0 software, with 1000 bootstrap replicates.

To investigate the classification and evolution of *NtWAK* genes, we used MEGA 7.0 to analyze the evolutionary relationships between 25 Arabidopsis *WAK* genes and 44 tobacco *WAK* genes (**Figure 2**). A phylogenetic tree was constructed, and based on their relatedness, the WAK proteins were classified into three groups. Group I comprised the largest group of WAK proteins, with 21 tobacco WAK proteins clustered alongside four Arabidopsis WAK proteins. Group II contained seven tobacco WAKs (NtWAK1, NtWAK5, NtWAK14, NtWAK15, NtWAK32, NtWAK33, NtWAK36) but harbored the highest number of Arabidopsis WAKs, totaling 15. Group III included 22 WAKs, 16 from tobacco, which clustered with six Arabidopsis WAK proteins. The phylogenetic analysis revealed that Arabidopsis WAKs did not intercalate with tobacco WAKs, indicating that *WAK* gene families likely underwent species-specific expansions following the divergence of tobacco and Arabidopsis, resulting in independent evolutionary trajectories.

### 3.3 Gene structure and conserved motif analysis of *NtWAK* genes

**Figure 3:**
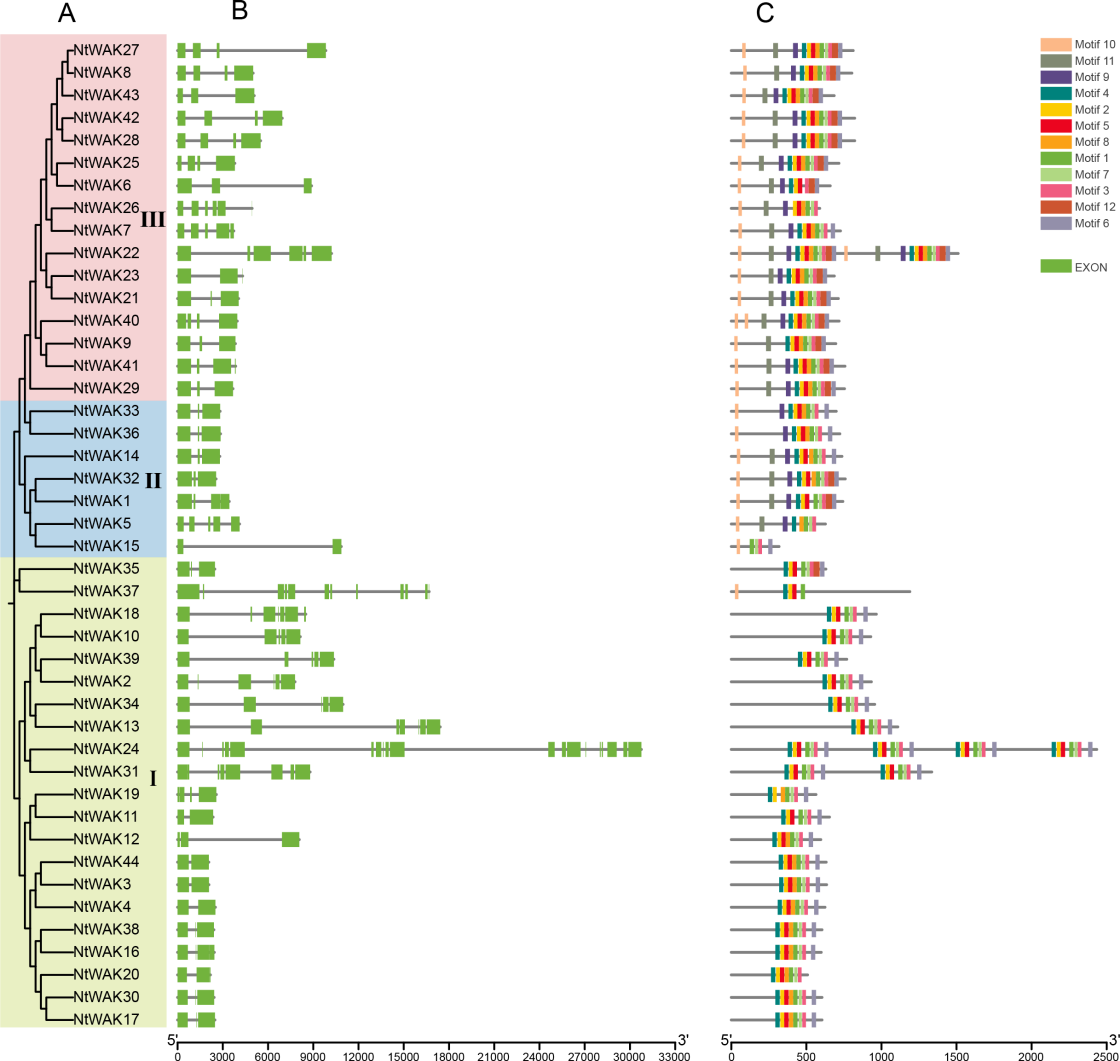
Gene structures and conserved motifs. **(A)** Phylogenetic tree of *NtWAK* genes. **(B)** Exons and introns structures of *NtWAK* genes. Green boxes represent exons, and black lines represent introns. **(C)** Conserved motifs of NtWAKs. Conserved motifs are represented by boxes in different colors.

To investigate the structural variation of tobacco *WAK* genes, we constructed a phylogenetic tree based exclusively on tobacco *WAK* genes, which was also classified into three distinct groups (**Figure 3A**). Exon-intron structural analysis of *NtWAK* genes revealed considerable variation in exon numbers, ranging from 2 to 19 exons across the family (**Figure 3B**). Genes within the same phylogenetic clade exhibited similar structural features, including comparable intron counts in their coding sequences. In contrast, genes from different groups exhibited considerable structural variation. These findings suggest that the evolution of the tobacco *WAK* gene family involved significant gains and losses of introns. To further explore the functional and structural properties of the tobacco *WAK* gene family, we utilized the MEME online tool and TBtools to identify and visualize conserved motifs across the NtWAKs (**Figure 3C**). A total of 12 conserved motifs were detected in 44 NtWAKs (**Supplementary Figure S2**). Notably, NtWAK15 and NtWAK37 contained the fewest motifs, with only five, whereas the remaining genes displayed between 8 and 12 motifs. Motifs 1 to 8 were commonly found across the majority of family members. Furthermore, all NtWAKs from Groups II and III possessed motif 10, while genes from Group I were generally devoid of this motif, except for NtWAK37. The observed structural and motif variations imply functional diversification within the tobacco *WAK* gene family.

### 3.4 Promoter analysis of *NtWAK* genes

**Figure 4:**
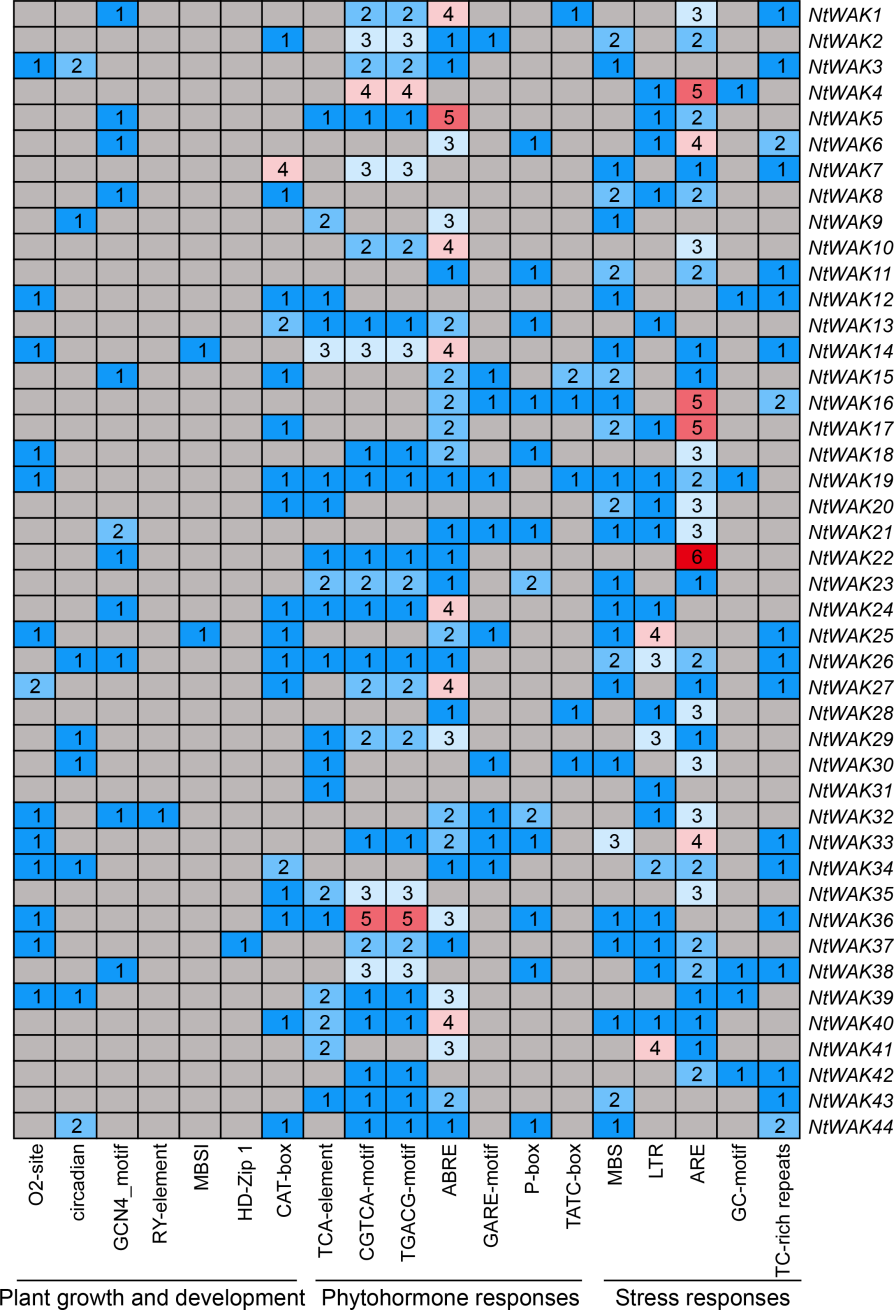
*Cis*-acting elements in the *NtWAK* gene promoters. The numbers within the colored boxes indicate the number of *cis*-acting elements in the promoters of the each *NtWAK* gene.

To investigate the potential functions of the tobacco *WAK* genes, we utilized the PlantCARE online tool to analyze the 2000 bp upstream promoter regions of *NtWAK* gene family members. The identified *cis*-acting elements, as illustrated in the figure, include numerous regulatory motifs associated with plant growth and development, phytohormone signaling, and stress responses (**Figure 4**; **Supplementary Table S3**). Within the category of growth and development, several *cis*-acting elements were identified, including the O2-site (involved in zein metabolism), circadian (regulating circadian control), GCN4_motif (associated with endosperm expression), RY-element (seed-specific regulation), MBSI (regulating flavonoid biosynthesis), HD-Zip 1 (linked to palisade mesophyll cell differentiation), and CAT-box (regulating meristem expression). The plant hormone response elements identified primarily include salicylic acid (SA) response elements (TCA-element), methyl jasmonate (MeJA) response elements (CGTCA-motif and TGACG-motif), abscisic acid (ABA) response elements (ABRE), and gibberellin (GA) response elements (GARE-motif, P-box, and TATC-box). Among these, the *cis*-acting elements associated with MeJA and ABA responses were the most prevalent, with 102 MeJA-responsive elements and 77 ABA-responsive elements predicted. Both MeJA and ABA are key regulatory signaling molecules involved in plant development and stress response processes (Schlögl et al., 2008). Moreover, several stress-related *cis*-acting elements were identified, including MBS (drought stress), LTR (low-temperature stress), ARE (anaerobic induction), GC-motif (anoxic-specific inducibility), and TC-rich repeats (involved in defense and stress responses). These findings indicate that members of the *NtWAK* gene family are involved in various aspects of tobacco growth and development, including growth regulation, phytohormone signaling, and responses to stresses.

### 3.5 Expression profiles of *NtWAK* genes

**Figure 5:**
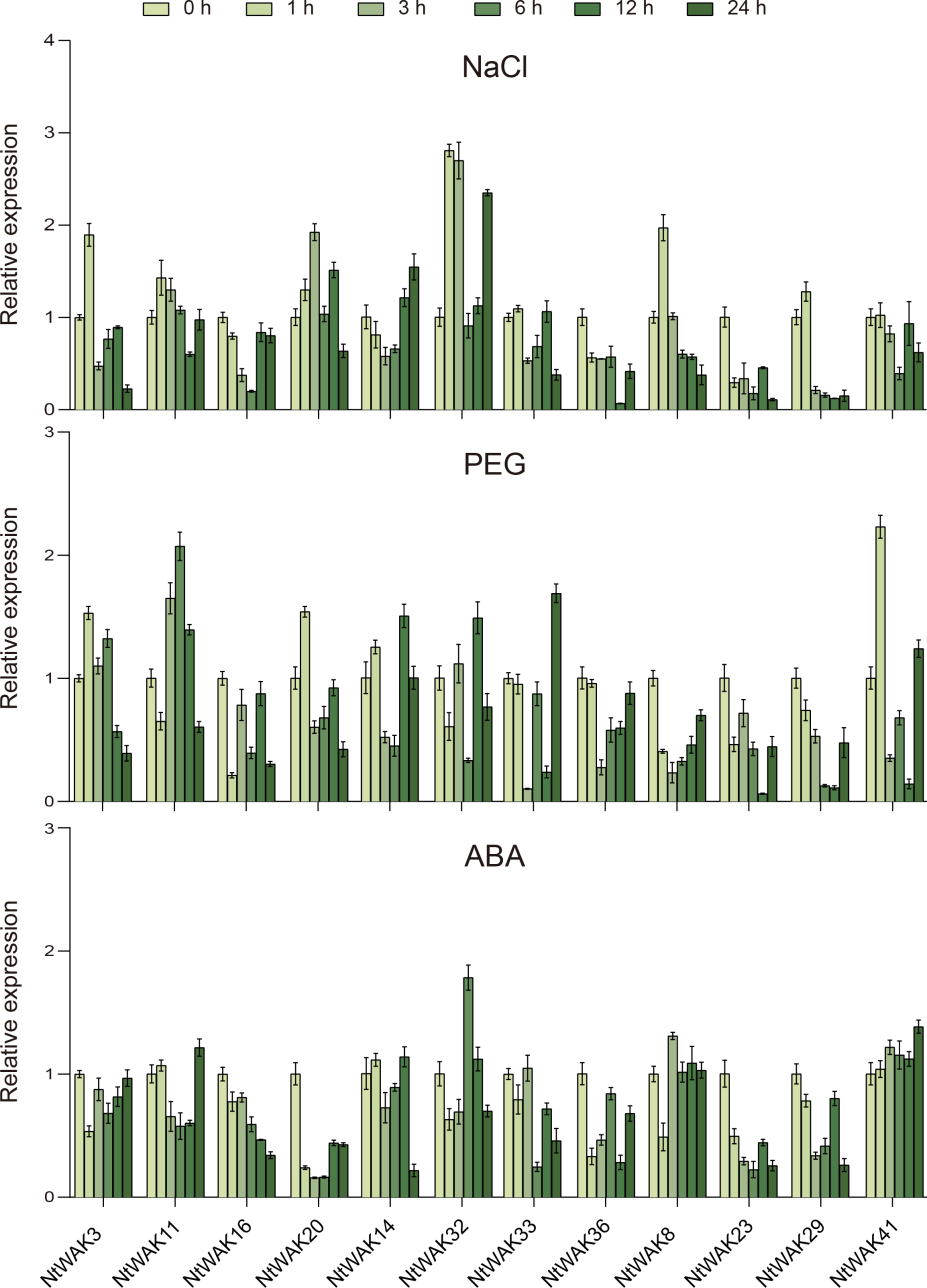
Expression patterns of *NtWAK* genes under NaCl, PEG, and ABA treatments. The *NtActin* gene was used as an internal control. The transcription levels of each gene were calculated using the 2^-ΔΔCt^ method, and the results are presented as the mean ± standard error, with n = 3.

To examine the expression patterns of *NtWAK* gene family members under abiotic stress, four genes were randomly selected from each of the three groups of the NtWAK family. Specifically, *NtWAK3*, *NtWAK11*, *NtWAK16*, and *NtWAK20* from Group I; *NtWAK14*, *NtWAK32*, *NtWAK33*, and *NtWAK36* from Group II; and *NtWAK8*, *NtWAK23*, *NtWAK29*, and *NtWAK41* from Group III were selected. Their expression patterns in response to salt, drought, and ABA treatments were then analyzed using qRT-PCR (**Figure 5**). Differential expression patterns of the *NtWAK* genes were observed in response to salt drought, and ABA stress. Notably, the genes *NtWAK16*, *NtWAK23*, and *NtWAK36* were not induced by any of these stress treatments. NaCl treatment initially induced the expression of several genes, including *NtWAK3*, *NtWAK8*, *NtWAK11*, *NtWAK20*, *NtWAK29*, and *NtWAK32*, followed by a subsequent down-regulation of their expression. Moreover, *NtWAK14* exhibited a pattern of initial down-regulation followed by up-regulation under salt treatment, with expression peaking 24 h post-treatment. In contrast, the transcriptions of *NtWAK33* and *NtWAK41* were inhibited. Under PEG-induced drought stress, the expression of *NtWAK8* and *NtWAK29* was consistently suppressed, while the expression of other genes was induced at varying time points. The ABA-induced stress response was the least pronounced among the three treatments, although ABA did promote the transcription of *NtWAK32* and *NtWAK41*. These results indicate that NtWAK family members are extensively involved in tobacco’s response to abiotic stresses, exhibiting diverse expression profiles under varying environmental conditions.

### 3.6 Subcellular localization and kinase activity of NtWAKs

**Figure 6:**
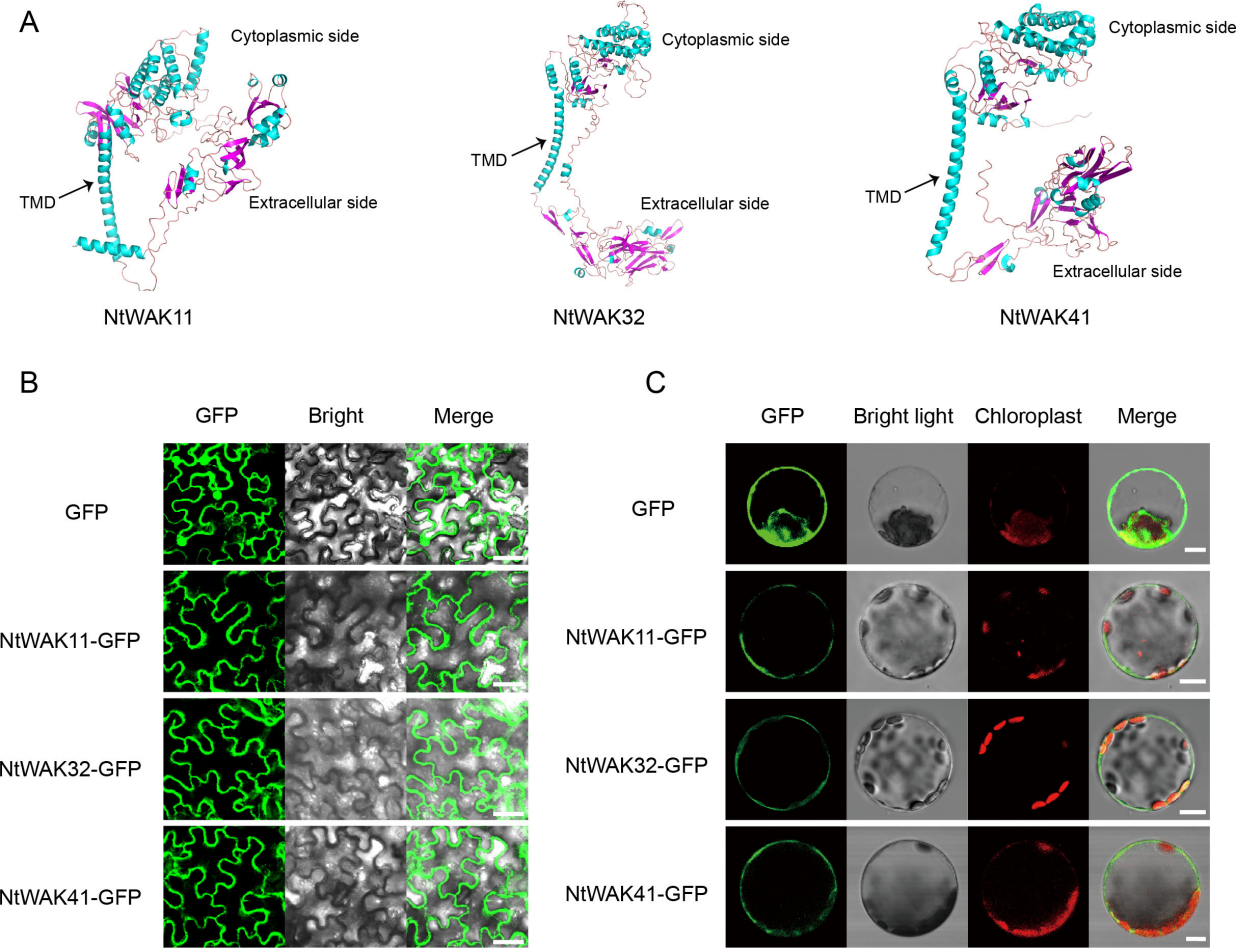
Three-dimensional models and subcellular localization. **(A)** Three-dimensional models of NtWAK11, NtWAK32, and NtWAK41. **(B)** Subcellular localization of NtWAK11, NtWAK32, and NtWAK41 in tobacco leaf cells. **(C)** Subcellular localization of NtWAK11, NtWAK32, and NtWAK41 in Arabidopsis protoplasts. Scale bar = 10 μm.

**Figure 7:**
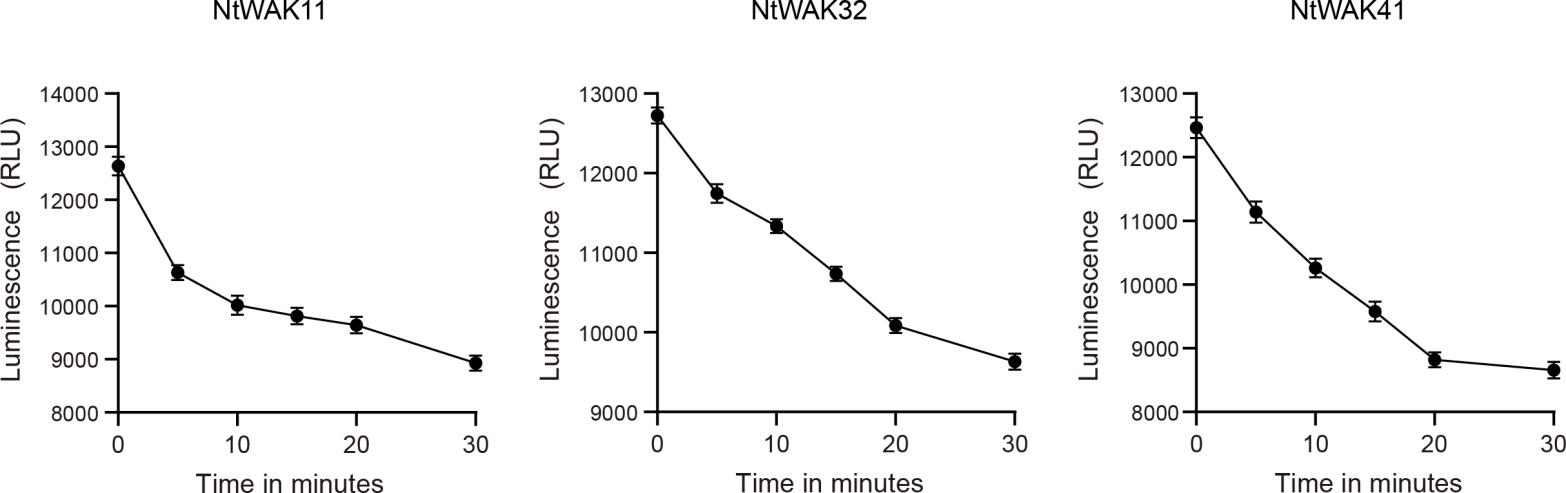
Kinase activity of WAK proteins *in vitro*. The luminescent signal is directly proportional to the ATP concentration in the solution and inversely correlated with the level of kinase activity.

According to the results of qRT-PCR, we selected one gene from each of the three groups, *NtWAK11*, *NtWAK32* and *NtWAK41*, whose expression levels changed significantly under abiotic stress. The three-dimensional structures and subcellular localizations of these genes were subsequently analyzed. Initially, the three-dimensional structures of the NtWAK proteins were predicted using homology modeling, and the analysis revealed that all three proteins contain multiple α-helices, β-sheets, and coil structures. While their intracellular structures exhibit similar conformations, distinct variations were observed in their extracellular domains (**Figure 6A**). Subsequently, to investigate the subcellular localization of the three NtWAKs, recombinant vectors for NtWAK11-GFP, NtWAK32-GFP, and NtWAK41-GFP were constructed and individually introduced into tobacco mesophyll cells (**Figure 6B**). The *p*CAMBIA2300-GFP empty vector displayed fluorescence in the cell membrane, nucleus, and cytoplasm. In contrast, the constructs NtWAK11-GFP, NtWAK32-GFP, and NtWAK41-GFP exhibited the strongest fluorescence in the plasma membrane. To exclude the potential influence of the cell wall, the recombinant proteins were transiently expressed in Arabidopsis protoplasts (**Figure 6C**). Consistent with the results observed in tobacco cells, green fluorescence was detected exclusively in the outer ring of the protoplasts. The *in vitro* kinase activity assay showed that with the increase in reaction time, the luminescent signal gradually weakened. This indicates a progressive reduction in the unreacted ATP within the system, suggesting that NtWAK11, NtWAK32, and NtWAK41 possess phosphorylation activity (**Figure 7**). These observations support the conclusion that NtWAK11, NtWAK32, and NtWAK41 are kinases with phosphorylation activity localized to the plasma membrane.

## 4 Discussion

WAK proteins are integral to cell wall dynamics, interacting with its components to detect structural alterations. Their kinase domain is responsible for initiating downstream signaling pathways, thereby facilitating cellular responses to these changes (Kanneganti and Gupta, 2008). WAKs serve diverse functions in the physiological regulation of plants, acting as a key mediator in cell wall signaling and facilitating adaptation to environmental changes (Kohorn, 2016; Stephens et al., 2022). In this study, 44 *WAK* genes were identified in the genome of *N. tabacum*, which is the same number observed in apple (Zuo et al., 2018). This count surpasses those identified in Arabidopsis (Verica and He, 2002), *N. benthamiana* (Zhong et al., 2023), tomato (Sun et al., 2020), and potato (Yu et al., 2022), but is lower than the numbers reported in rice (Zhang et al., 2005), barley (Tripathi et al., 2021), and rose (Liu et al., 2021). The evolutionary analysis of *WAK* genes indicates that the *WAK* gene family has rapidly expanded in angiosperms (Zhang et al., 2023), and the differences in gene number may reflect the evolutionary relationships between species. By conducting sequence comparisons and phylogenetic analysis with Arabidopsis WAK proteins, 44 *NtWAK* genes were classified into three distinct groups. Phylogenetic analysis revealed that tobacco *WAK* genes clustered into several tobacco-specific branches, suggesting a lineage-specific expansion of the *WAK* gene family following the divergence of tobacco. This observation is consistent with previous studies on cotton and *N. benthamiana* (Zhang et al., 2021; Zhong et al., 2023), which demonstrated species-specific expansion of *WAK* genes following speciation.

The structure of exons and introns is not only a fundamental component of the gene expression process but also highlights the adaptability and diversity inherent in genomic evolution (Shaul, 2017). In this study, we conducted a structural analysis of the *NtWAK* gene and identified distinct exon-intron organization patterns in *NtWAK* genes distributed across different phylogenetic groups. The number of exons in the *NtWAK* gene varied from 2 to 19, potentially reflecting intron gain and loss events that occurred as an adaptive response to environmental pressures over the course of long-term evolution. The analysis of conserved protein motifs offers valuable insights into the relationships between protein similarity, structure, and function. Analysis of NtWAK protein motifs revealed that proteins within the same evolutionary clade share similar types and distributions of conserved motifs, while differences among subgroups are primarily localized at the N-terminus. This finding aligns with the observation that the cytoplasmic region of Arabidopsis WAKs exhibit higher sequence identity compared to its extracellular region (Verica et al., 2003), supporting the notion that WAK perceives diverse external signals through distinct extracellular domains (Kanneganti and Gupta, 2008).

*Cis*-acting elements within promoters play a central role in regulating gene expression by interacting with transcription factors and other proteins, thereby modulating gene activation, expression levels, and spatiotemporal specificity (Hernandez-Garcia and Finer, 2014). To explore the potential regulatory mechanisms governing the expression of *NtWAK* genes, this study analyzed the *cis*-acting elements within the promoter region of *NtWAK* genes and counted 19 elements associated with plant growth and development, phytohormone signaling, and stress responses. The promoter regions of *NtWAK* genes contained numerous *cis*-acting elements linked to responses to SA, MeJA, ABA, and GA, with a particular abundance of elements associated with MeJA and ABA signaling. Plant hormones serve as essential signaling molecules, orchestrating various processes related to plant growth, development, and defense mechanisms (Shan et al., 2012). Furthermore, several *cis*-acting elements related to stress responses were identified within the promoter regions of *NtWAK* genes, such as MBS, LTR, ARE, GC-motif, and TC-rich repeats. The diversity of *cis*-acting elements in the promoter region of *NtWAK* genes indicates that *NtWAK* genes may participate in multiple biological processes and play a multifunctional regulatory role in the normal growth of tobacco and the response to environmental stress.

Numerous studies have highlighted the critical role of *WAK* genes in mediating responses to abiotic stress. Pepper CaWAKL20 acts as a negative regulator of plant thermotolerance by suppressing the expression of ABA-responsive genes (Wang et al., 2019). Rice OsWAK112 likely negatively regulates plant salt tolerance by suppressing ethylene biosynthesis via its direct interaction with S-adenosyl-L methionine synthetase (SAMS) 1/2/3 (Lin et al., 2021). In this study, we investigated the dynamic expression profiles of 12 *NtWAK* genes under conditions of salt stress, drought, and ABA treatment. The results showed that, except for *NtWAK16*, *NtWAK23*, and *NtWAK36*, which were down-regulated under all three stress conditions, the remaining genes exhibited dynamic expression patterns. Furthermore, individual *NtWAK* genes within the same subgroup displayed varying expression patterns under the same stress conditions, indicating that they may exhibit distinct regulatory properties. Three-dimensional structural analysis of three subgroups of NtWAK proteins showed that they have a high degree of structural similarity in one side of the cytoplasm. This was also consistent with the distribution of conserved motifs. Subcellular localization analysis revealed that the three NtWAK proteins are localized to the plasma membrane, which is consistent with findings in other species (Lin et al., 2021; Zhang et al., 2021), suggesting their significant role in the transmembrane transmission of environmental signals.

## 5 Conclusions

This study comprehensively identified and analyzed members of the *WAK* gene family in *N. tabacum*. A total of 44 *NtWAK* were identified and the phylogenetic tree divided them into three subgroups. Phylogenetic analysis revealed that the *NtWAK* genes could be classified into three subgroups, and a species-specific expansion of the *WAK* gene family occurred during tobacco evolution. Analysis of gene structure and conserved motifs suggested potential functional diversification among the *NtWAK* genes. *Cis*-acting elements in the promoter regions indicated their involvement in various physiological processes. Expression profiling demonstrated that *NtWAK* genes were induced by different abiotic stresses. These findings not only lay a foundation for further exploration of the functional mechanisms of *WAK* genes but also provide new theoretical insights for enhancing stress resistance and guiding the genetic improvement of tobacco.

## Data Availability

The original contributions presented in the study are included in the article/**Supplementary Material**. Further inquiries can be directed to the corresponding authors.

## Supplementary material

Supplementary Figure 1 Protein structures of NtWAKs.

Supplementary Figure 2 Conserved motifs of NtWAKs.
