# Supplementary figures and images for: Identification and functional analysis of Wall-Associated Kinase genes in Nicotiana tabacum
Source: Front Plant Sci. 2025 Feb 5;16:1543437. doi: 10.3389/fpls.2025.1543437 (PMC11835679; doi:10.3389/fpls.2025.1543437)

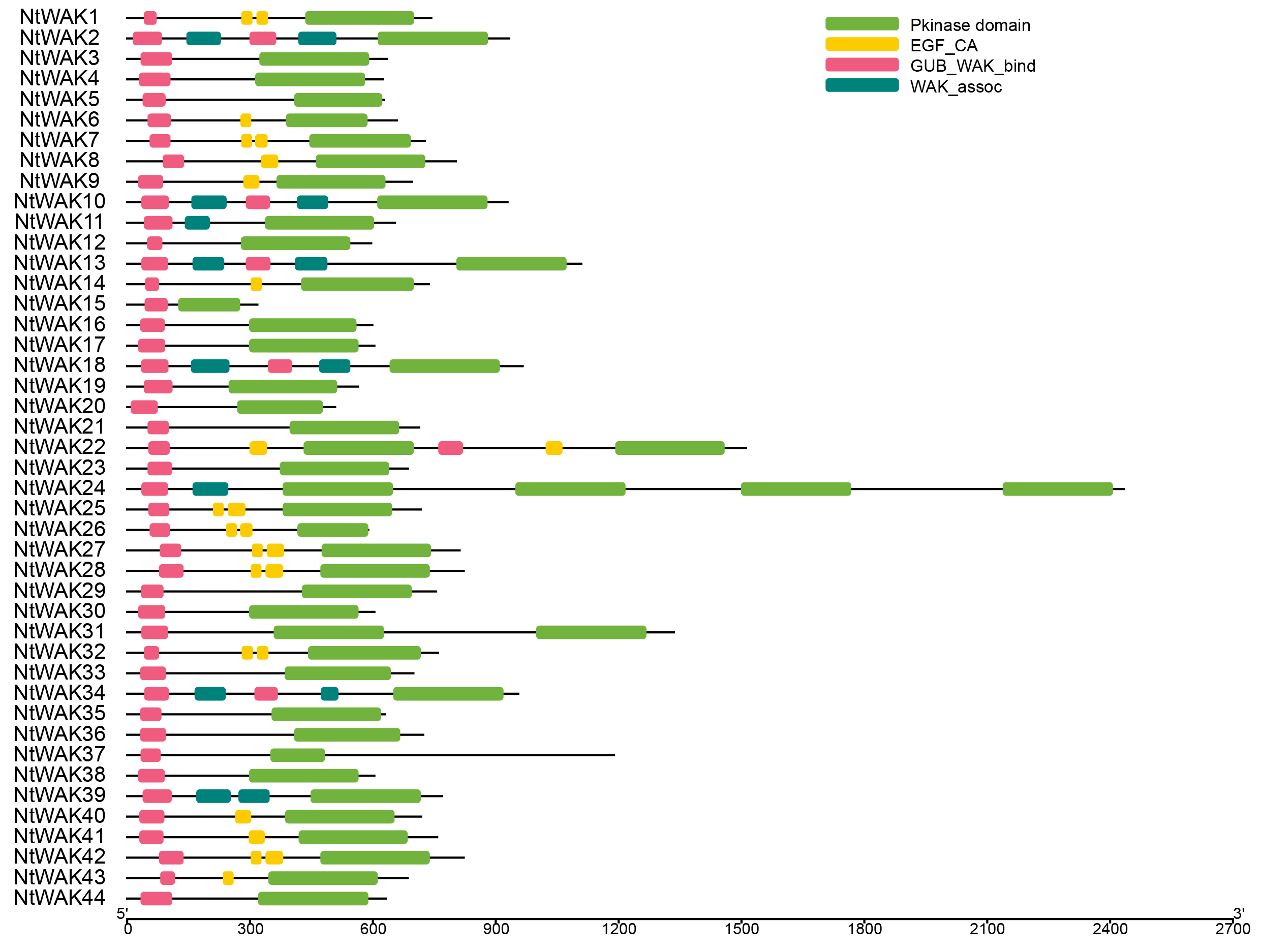

Supplement: Supplementary Figure 1 — Protein structures of NtWAKs. [file Image1.tif]

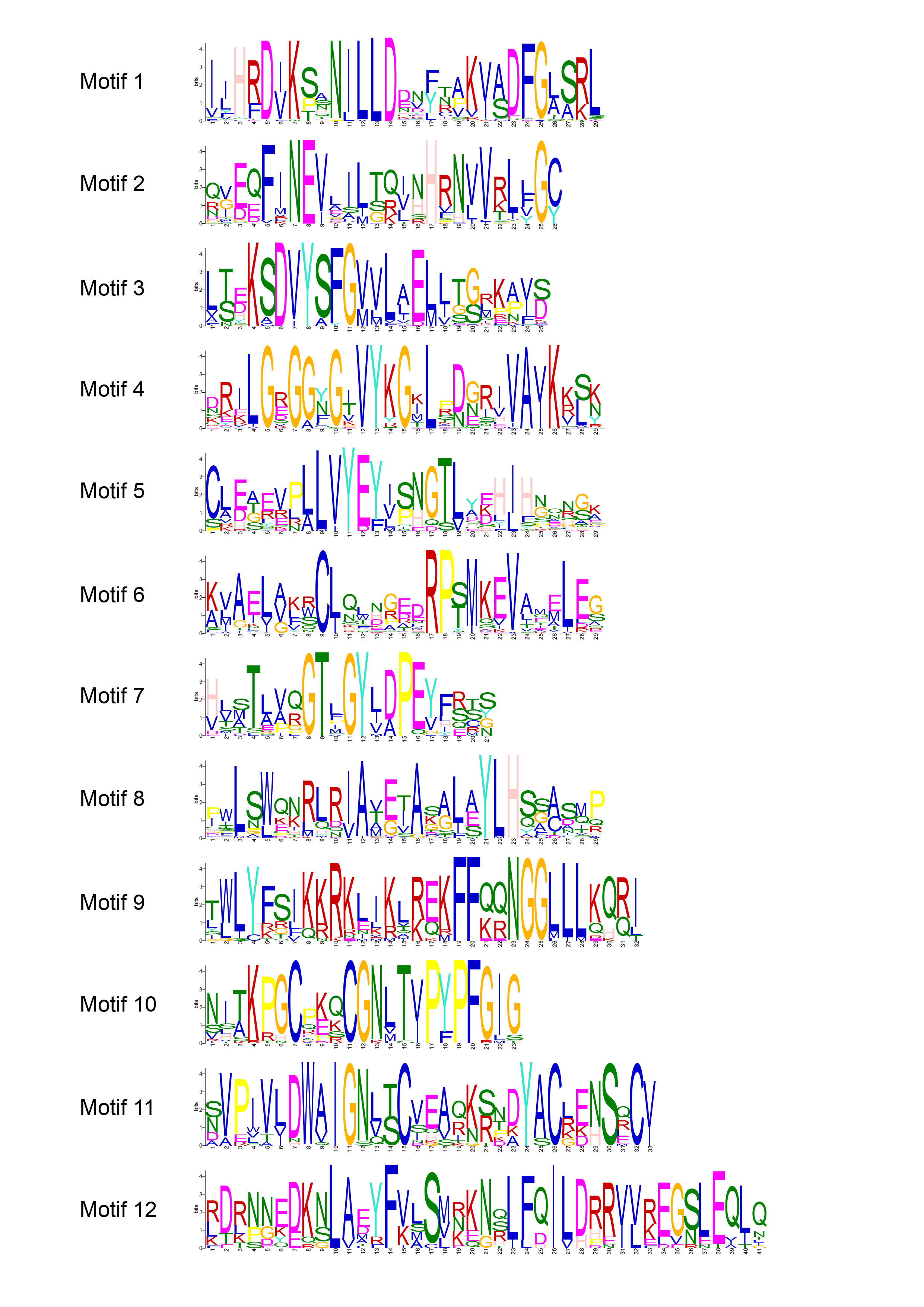

Supplement: Supplementary Figure 2 — Conserved motifs of NtWAKs. [file Image2.tif]
